# PLA2G7 promotes immune evasion of bladder cancer through the JAK-STAT-PDL1 axis

**DOI:** 10.1038/s41419-025-07593-1

**Published:** 2025-04-01

**Authors:** Ding Peng, Wuping Yang, Tianyu Tang, Anbang He, Xin Xu, Taile Jing, Dan Xia

**Affiliations:** Department of Urology, The First Affiliated Hospital, School of Medicine, Zhejiang University, 79 Qingchun Road, Hangzhou, Zhejiang Province 310003 PR China

**Keywords:** Bladder cancer, Cancer immunotherapy

## Abstract

Targeting immune checkpoints such as Programmed death ligand-1 (PD-L1) and Programmed cell death 1 (PD-1) has been approved for treating bladder cancer and shows promising clinical benefits. However, the relatively low response rate highlights the need to seek an alternative strategy to traditional PD-1/PD-L1 targeting immunotherapy. In this study, we found that PLA2G7 is significantly elevated in bladder cancer and correlates with worse prognosis. In vitro experiments demonstrated that knockdown of PLA2G7 does not significantly affect the proliferation, migration, and invasion of bladder cancer cells. Flow cytometry detection, as well as protein and RNA detection, showed that knockdown of PLA2G7 significantly inhibits PD-L1 expression and suppresses the growth of transplanted tumors by promoting CD8 + T-cell infiltration. Further experiments showed that PLA2G7 regulates the JAK-STAT pathway to promote PD-L1 expression by upregulating the phosphorylation of STAT1 and STAT3. Meanwhile, results from syngeneic mouse models indicated that PLA2G7 suppression and anti-CTLA4 therapy have synergistic effects on tumor burden and mouse survival. In addition, we found that ETS1 promotes PLA2G7 overexpression in bladder cancer cells. In summary, our findings provide a novel immunotherapeutic strategy against bladder cancer through targeting the ETS1-PLA2G7-STAT1/STAT3-PD-L1 axis.

## Introduction

Bladder cancer is one of the most common cancers of the urinary system. According to statistics, there are more than 570,000 new cases of bladder cancer and more than 200,000 deaths from bladder cancer each year [1]. Approximately 75% of patients with bladder cancer is non-muscle-invasive bladder cancer (NMIBC), and these patients have a high rate of recurrence and progression after transurethral resection [2]. Tumors that invade the muscle layer of the bladder are called muscle-invasive bladder cancer (MIBC), and these tumors are highly susceptible to spread and metastasis [3]. For patients with MIBC, radical cystectomy and combined therapy based on chemotherapy and radiotherapy are the main treatments [4]. In recent years, the emergence of immunotherapy has provided a new therapeutic method for the treatment of bladder cancer. However, due to the individual heterogeneity of bladder cancer, immunotherapy is not effective for some patients, and only a subset of patients can benefit from immune checkpoint inhibitors (ICIs) such as programmed cell death-1/programmed death-ligand 1 (PD-1/PD-L1) antibody immunotherapy [4]. Therefore, it is of great importance to further explore the molecular mechanisms of immune escape and immunotherapy resistance in bladder cancer and to identify novel therapeutic targets. In fact, continuous research has advanced the progress of new therapeutic targets and monitoring indicators for bladder cancer in recent years [5–8].

Phospholipase A2 Group 7 (PLA2G7), also known as lipoprotein-associated phospholipase A2 or platelet-activating factor acetylhydrolase, is a phospholipase involved in the hydrolysis of platelet-activating factor and truncated phospholipids synthesized through oxidation [9]. Previous studies have found that PLA2G7 is involved in the development of a variety of diseases, including atherosclerosis, diabetes, and autoimmune diseases [10–13]. Furthermore, investigations have also found that PLA2G7 is involved in the development of multiple tumors [14–20] and the PLA2G7 inhibitor darapladib plays a significant inhibitory role in a variety of tumors by sensitizing cancer cells to ferroptosis [21, 22]. Moreover, recent studies have found that PLA2G7 also plays an important role in tumor immunity. For example, in hepatocellular carcinoma, PLA2G7 promotes cancer cell development through the STAT1/PD-L1 axis, and high expression of PLA2G7 in macrophages can promote immunosuppression by adversely affecting the activation of CD8+ T cells [23, 24].

However, the role of PLA2G7 in bladder cancer and tumor immunity is still unclear. This study revealed that PLA2G7 regulates the JAK-STAT pathway to promote PD-L1 expression and immune evasion in bladder cancer. Targeting this pathway could be a novel therapeutic strategy for bladder cancer.

## Materials and methods

### Human tissue specimens

Bladder cancer and adjacent normal tissues were obtained from the First Affiliated Hospital, College of Medicine, Zhejiang University. The ethics committees of the First Affiliated Hospital, School of Medicine, Zhejiang University, approved the protocol.

### Cell culture and reagents

Bladder cancer cell lines 5637, T24, and MBT-2 were cultured in RPMI-1640 medium (Gibco, USA). All media contained 10% fetal bovine serum (Gemini, USA), penicillin G (100U/ml), and streptomycin (100 µg/ml) (Sigma-Aldrich, Germany). Cells were maintained as monolayer cultures at 37 °C in a humidified atmosphere containing 5% CO_2_. The antibodies used in this study were as follows: PLA2G7 (Proteintech, 15526–1-AP), JAK2 (Abcam, ab108596), STAT3 (Abcam, ab68153), p-STAT3 (Abcam, ab76315), GAPDH (Proteintech, 60004-1-Ig), STAT1 (Proteintech, 10144-2-AP), p-STAT1 (Cell Signaling Technology, 9167), IRF1 (Proteintech, 11335-1-AP), PD-L1 (Proteintech, 66248-1-Ig), ETS1 (Proteintech, 66598-1-Ig), CD4 (Abcam, ab237722), and CD8 (Abcam, ab217344).

### Cell transfection

PLKO.1 vector plasmids were purchased from BioVector NTCC, Inc. (Guangzhou, China). Cells were cultured for 24 h prior to transfection and were then transiently transfected with the corresponding shRNA vector using Lipofectamine 3000 Transfection Reagent (Invitrogen, Carlsbad, CA, USA) according to the manufacturer’s instructions. Supplementary Table S1 shows the detailed shRNA sequences used in this study. For the overexpression of PLA2G7, recombinant pGC-LV-GV287-GFP vectors containing either PLA2G7 mRNA or a scrambled control sequence were constructed by Genechem Company (Shanghai, China). Briefly, pGC-LV-GV287-GFP-PLA2G7 or a control vector mixed with pHelper1.0 and pHelper2.0 was co-transfected into HEK-293T cells using PEI (Sigma-Aldrich). Lentivirus-mediated infection of cells was performed according to the manufacturer’s recommended MOI value. OE-PLA2G7 and OE-CTRL cells were selected with neomycin (200 µg/mL). Transfection efficiency was validated by Western blot.

### Immunohistochemical analysis

Tissues were fixed with 4% formalin and embedded in paraffin wax before being cut into 5-µm sections on a microtome. Sections were deparaffinized in xylene and rehydrated with graded concentrations of alcohol. Subsequently, the slides were treated with 3% H_2_O_2_ to block endogenous peroxidase activity and were heated at 95 °C for 2.5 min in citrate buffer (10 mM, pH 6.0) for antigen retrieval. To reduce nonspecific binding, 10% normal goat serum was applied. The slides were then incubated with primary antibodies at 4 °C overnight. A Power-Vision™ two-step histostaining reagent and 3,3’-diaminobenzidine tetrahydrochloride substrate kit (ZSGB-Bio, China) were used to visualize the antigen according to the manufacturer’s instructions.

### MTS cell viability assays

Cell viability assays were evaluated using CellTiter 96 AQ One Solution Reagent (Promega, USA) in accordance with the manufacturer’s instructions.

### RT-qPCR

Total RNA was isolated from cell lines and tissues using TRIzol reagent. Two μg of RNA was reverse transcribed into cDNA using M-MLV reverse transcriptase. Quantitative PCR was carried out using SYBR^®^ FAST qPCR Kits with a final volume of 10 μl and using the 7500 Fast Real-Time PCR System. Target mRNA expression was normalized to GAPDH. Supplementary Table S2 shows the detailed sequences of the PCR primers included in this study.

### Western blot analysis

Total protein was isolated from cells using ice-cold radioimmunoprecipitation assay buffer and quantified with the BCA protein assay reagent. Equal amounts of protein were separated by SDS-PAGE and transferred to polyvinylidene fluoride membranes. After blocking with 5% milk for 1 h, the membranes were incubated with primary antibodies overnight at 4 °C. The signals were then detected after washing and incubation with secondary antibodies using ECL Western blotting detection reagents.

### Mouse xenograft model experiment

In the mouse xenograft experiment, MBT-2 cells were suspended in 100 µl of Matrigel matrix diluted 1:1 with PBS and subcutaneously injected into the axillary fossae of BALB/C or NOD-SCID mice (6–8 weeks old). Once tumors were palpable, tumor volume was measured and monitored throughout the study. Tumor volume was calculated using the following formula: V = 0.52 × *a* × *b*^2^, where *a* is the longest tumor axis and *b* is the shortest axis of the tumor. When the tumor reached about 100 mm^3^, mice were randomly divided into the control and treat groups. In the anti-PD-L1 immunotherapy model, mice received intraperitoneal injections of 200 µg anti-PD-L1 antibody every other day. Animal ethics approval for the animal protocol was obtained from the Animal Ethics Committee of the First Affiliated Hospital, School of Medicine, Zhejiang University.

### Flow cytometry analysis

Mouse tumors were cut into small pieces, disaggregated with collagenase, and filtered through 70-µm strainers. Cells were incubated with purified antibodies for 30 min at 4 °C for membrane staining according to the manufacturer’s instructions. Detection and analysis of stained samples were performed using eight-color flow cytometry (BD Biosciences).

### RNA-seq and data analysis

The KAPA Stranded RNA-seq Library Preparation Kit was used to construct RNA-seq libraries according to the manufacturer’s instructions. Sequencing reads were aligned to the hg19 human genome using the TopHat program (TopHat v2.1.1) with default parameters. Total read counts for each protein-coding gene were extracted using HTSeq (HTSeq version 0.6.0) and then loaded into DEseq2 in R to calculate differentially expressed genes with FDR < 0.05.

### Transwell assays

For the Transwell assay, the upper chambers were coated with Matrigel (Corning, NY, USA). Cells were seeded in the upper chamber at a density of 2 × 10^4^ cells/well in culture medium containing 1% FBS, while the lower chambers contained culture medium with 20% FBS. Cells that invaded the bottom surfaces of the chambers were incubated for 24 h, fixed in 4% paraformaldehyde (Solarbio), stained with 1% crystal violet (Solarbio), and quantified by counting four random fields of view.

### Wound healing assay

For wound healing assays, stably transfected cells were seeded into 6-well plates, and an artificial wound was created using a 1000-µL pipette tip when the cells reached 90% confluence. The cells were then cultured in medium containing 1% FBS for 24 h. Images were taken to record the wound width at 0 and 24 h. Cell migration ability was evaluated by comparing the wound widths of different groups at 0 and 24 h.

### T cell mediated tumor cell killing assay

The medium used for T cell culture was RPMI1640 supplemented with 10% FBS, 100 U/mL penicillin and streptomycin, 100 IU/mL recombinant IL2, 2 mg/mL CD3 antibody, and 1 mg/mL CD28 antibody. After stimulation for 1 week, T cells were harvested and co-cultured with cancer cells. Cells were incubated for 48 h, followed by removal of cellular debris, and then harvested and labeled with annexin V and PI for fluorescence-activated cell sorting (FACS) analysis.

### Bioinformatic analysis

The clinical and expression data for bioinformatic analysis were downloaded from The Cancer Genome Atlas (TCGA) database (https://cancergenome.nih.gov).

### Statistical analysis

A minimum of three independent replicates were performed for all statistical analyses. Data are shown as mean ± sd and were analyzed by GraphPad Prism. A two-sided Student’s *t* test was used to determine statistical significance for two group comparisons. In all figures, the level of significance is reported as follows: NS > 0.05, *P < 0.05, **P < 0.01, and ***P < 0.001.

## Results

### PLA2G7 is highly expressed in bladder cancer

**Fig. 1:**
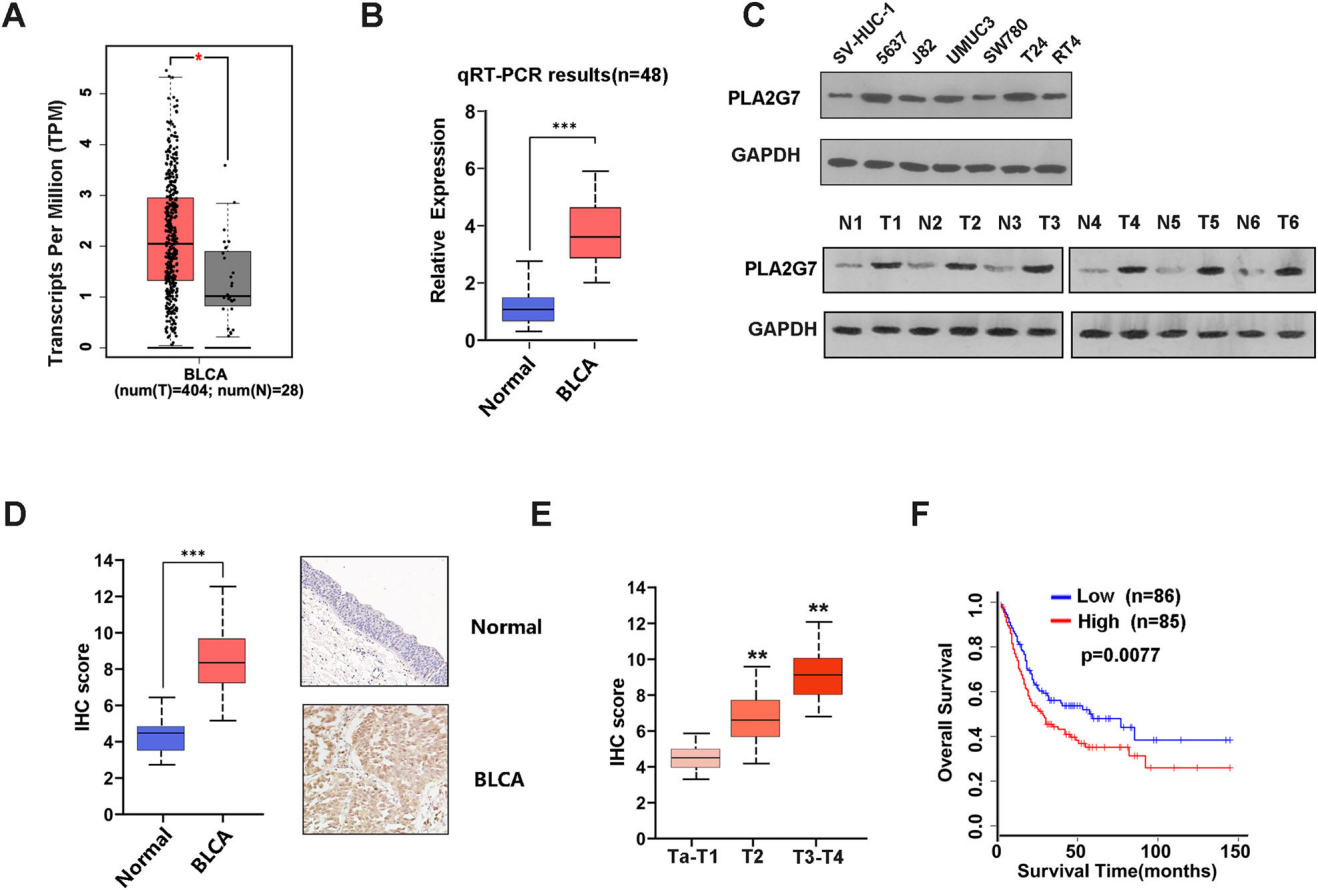
Elevated PLA2G7 expression is correlated with poor clinical outcome of bladder cancer. **A** The level of PLA2G7 mRNA in bladder cancer tumors compared to matched normal tissues (TCGA cohort). **B** The level of PLA2G7 mRNA in bladder cancer tumors compared to matched normal tissues (qRT-PCR result). **C** Western blot analysis showing PLA2G7 protein levels in normal bladder cell line compared bladder cancer cell lines, and bladder cancer tissues compared to matched normal tissues. **D** IHC results showing the protein level of PLA2G7 in bladder cancer tumors compared to matched normal tissues. **E** The level of PLA2G7 protein in different stage bladder cancer tissues. **F** Kaplan–Meier survival analysis of bladder cancer patients by different levels of PLA2G7 protein in tumors.

We first analyzed PLA2G7 expression in bladder cancer using data from the TCGA database and found that PLA2G7 is elevated in bladder cancer compared to normal bladder tissues (Fig. 1A). Overexpression of PLA2G7 in bladder cancer was also observed in RT-qPCR results (Fig. 1B). Additionally, Western blot analysis of bladder cancer and normal tissues as well as normal bladder cell line and bladder cancer cell lines revealed elevated PLA2G7 expression in bladder cancer (Fig. 1C). Immunohistochemical testing indicated that PLA2G7 is significantly overexpressed in bladder cancer, and increased PLA2G7 expression is significantly associated with higher tumor stage (Fig. 1D, E). Kaplan–Meier survival analysis showed that elevated PLA2G7 expression is significantly associated with worse prognosis (Fig. 1F).

### PLA2G7 did not affect bladder cancer cell proliferation, migration, and invasion

**Fig. 2:**
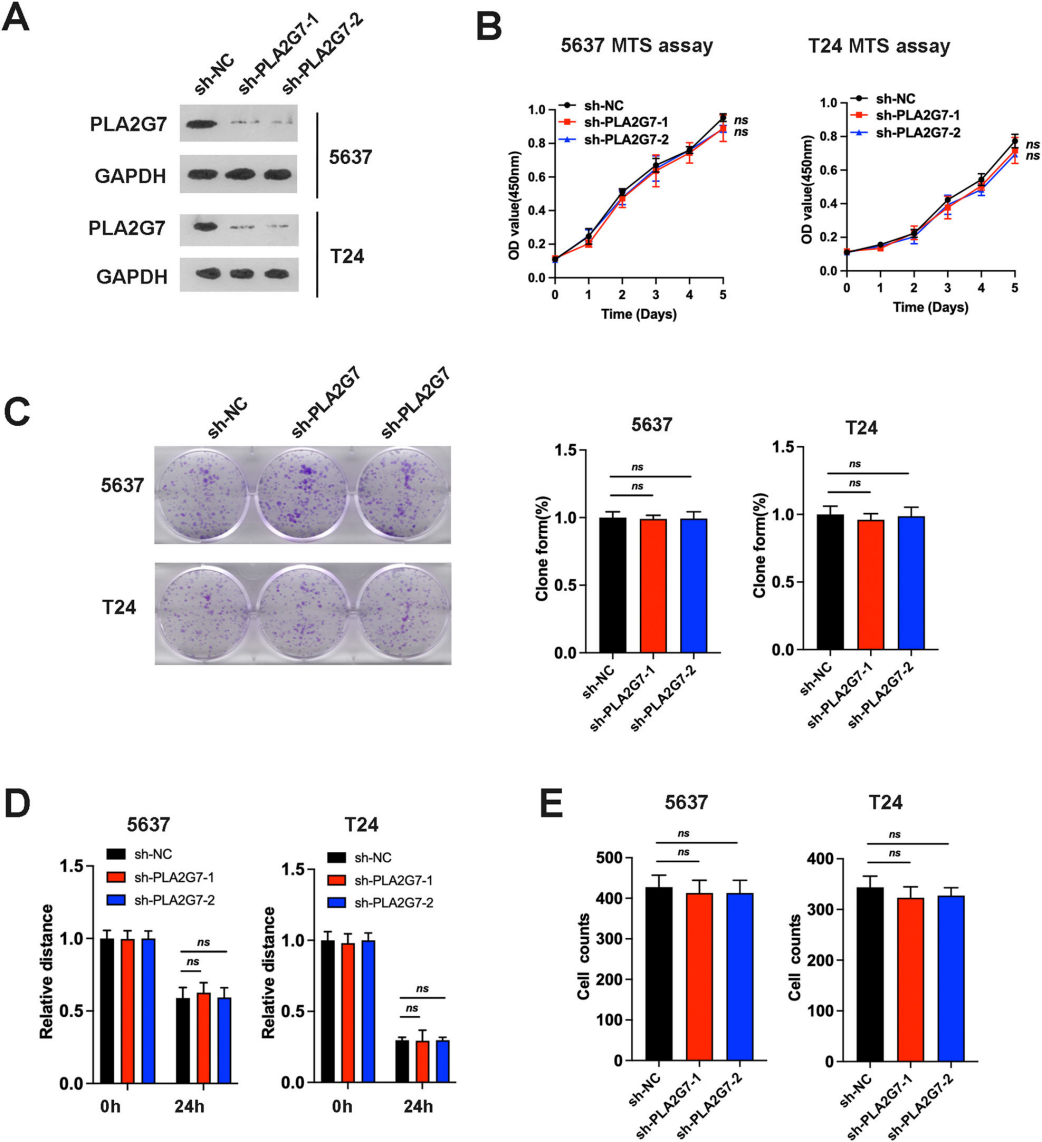
PLA2G7 does not affect bladder cancer cell proliferation, migration, and invasion. **A** Western blot analysis demonstrating the quality of PLA2G7 knockdown in 5637 and T24 cells. **B** MTS assays of sh-NC and sh-PLA2G7 cell viability. **C** Clone formation assays for sh-NC and sh-PLA2G7 cells. **D** Wound healing assays for sh-NC and sh-PLA2G7 cells. **E** Transwell assays for sh-NC and sh-PLA2G7 cells.

To further investigate the role of PLA2G7 in bladder cancer, we constructed stable PLA2G7 knockdown 5637 and T24 cell lines (Fig. 2A). MTS assay results indicated that PLA2G7 knockdown did not significantly affect bladder cancer cell proliferation (Fig. 2B). In clone formation experiments, PLA2G7 knockdown cells showed no significant difference compared to control cells (Fig. 2C). Wound healing and transwell assays also demonstrated that PLA2G7 knockdown did not significantly affect bladder cancer cell migration and invasion (Fig. 2D, E and Supplementary Fig. S1). These results suggest that PLA2G7 does not significantly impact the proliferation, migration, and invasion of bladder cancer cells.

### PLA2G7 depletion suppresses bladder cancer immune evasion

**Fig. 3:**
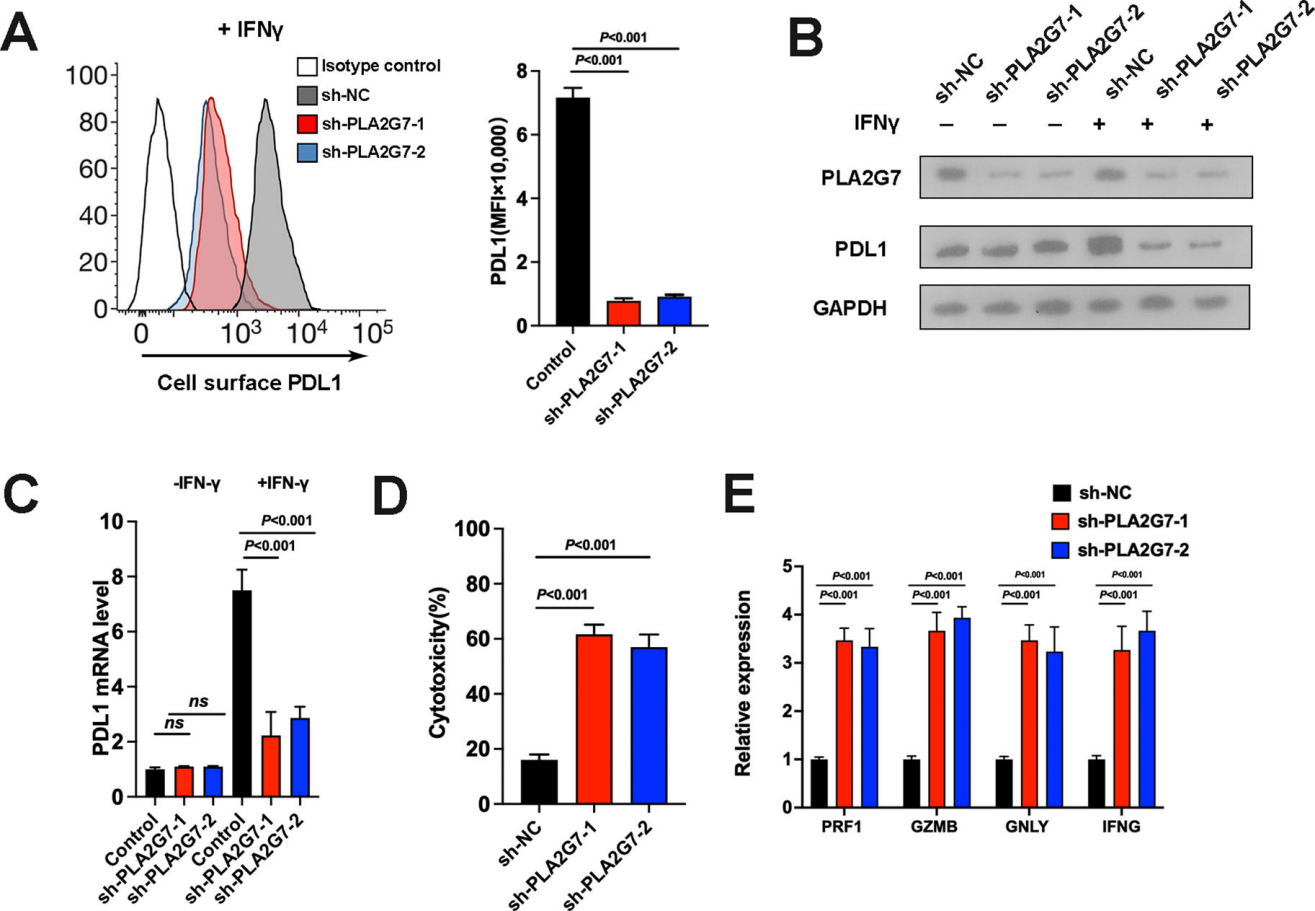
PLA2G7 depletion inhibits PD-L1 expression and tumor immune evasion. **A** Surface PD-L1 protein levels analyzed by FACS in sh-NC and sh-PLA2G7 cells. **B** Western blot analysis showing the levels of PD-L1 in sh-NC and sh-PLA2G7 cells with or without IFN-γ. **C** RT-qPCR analysis of the mRNA levels of PD-L1 in sh-NC and sh-PLA2G7 cells with or without IFN-γ. **D** Results from the T cell killing assays of sh-NC and sh-PLA2G7 cells. **E** Results from the expression of T cell activity factors including PRF1, GAMB, GNLY and IFNG of T cells that cocultured with sh-NC and sh-PLA2G7 cells.

Immune evasion is a crucial mechanism in tumor development, and the interaction between PD-1 and PD-L1 inhibits the activation of antigen-specific CD8+ T cells, aiding cancer cells in evading immune destruction. We next explored the effect of PLA2G7 on bladder cancer tumor immunity and PD-L1 expression. Using FACS analysis, we found that PLA2G7 depletion significantly inhibits PD-L1 expression (Fig. 3A). In the absence of IFN-γ, there was no significant difference in PD-L1 protein and mRNA levels between PLA2G7 knockdown and control cells. However, with IFN-γ stimulation, PD-L1 protein and mRNA levels were significantly decreased in PLA2G7 knockdown cells compared to control cells (Fig. 3B, C). T cell co-culture experiments demonstrated that PLA2G7 knockdown significantly enhanced T cell cytotoxicity (Fig. 3D). Detection of T cell activity factors also revealed that T cells co-cultured with PLA2G7 knockdown cells had significantly upregulated activity (Fig. 3E).

**Fig. 4:**
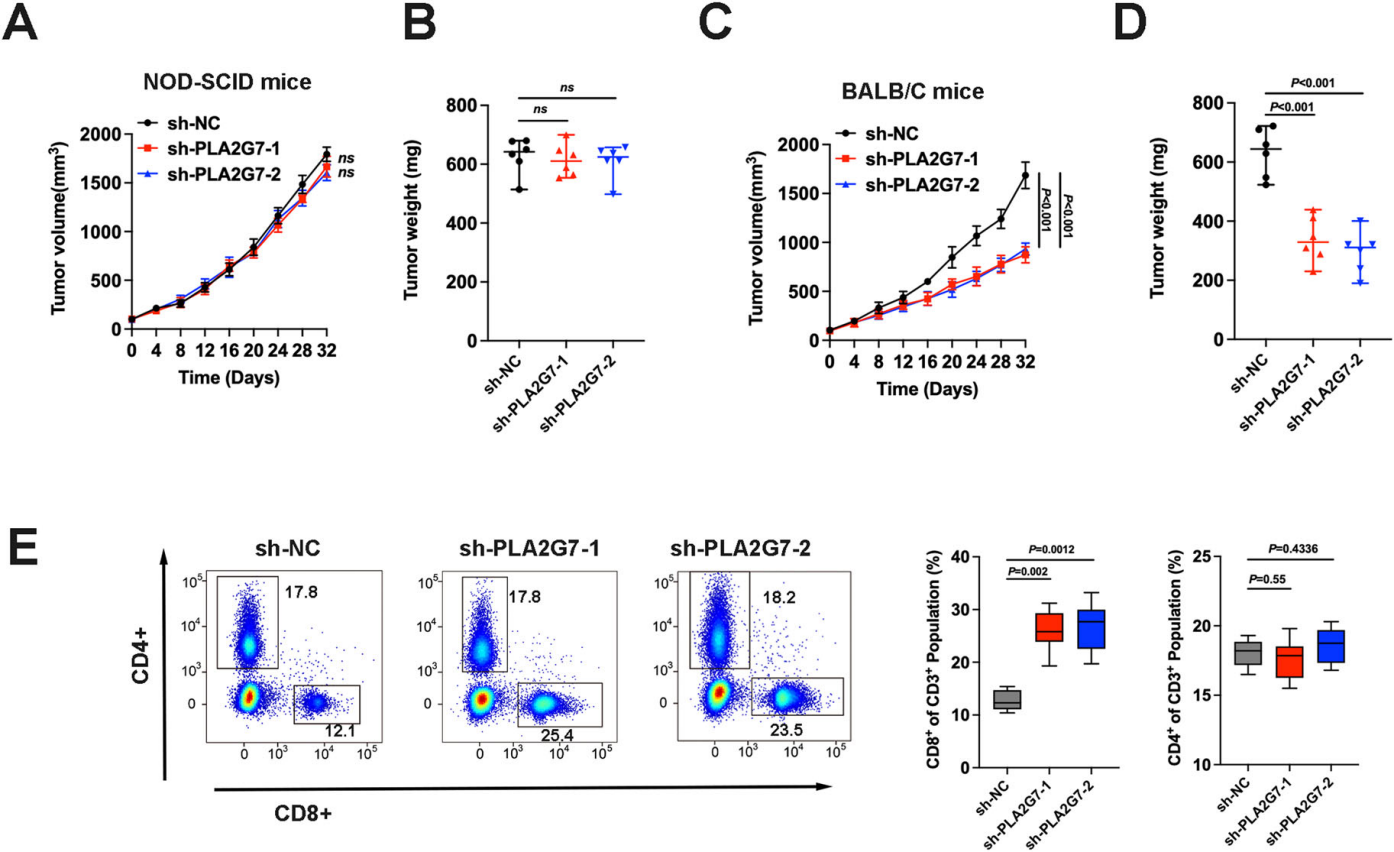
In vivo experiments in mice confirmed that PLA2G7 knockdown inhibited tumor immune escape. **A** Tumor growth curves for immunodeficient NOD-SCID mice injected with sh-NC and sh-PLA2G7 cells. **B** Tumor weights for immunodeficient NOD-SCID mice injected with sh-NC and sh-PLA2G7 cells. **C** Tumor growth curves for immunocompetent BALB/C mice injected with sh-NC and sh-PLA2G7 cells. **D** Tumor weights for immunocompetent BALB/C mice injected with sh-NC and sh-PLA2G7 cells. **E** Flow cytometry analysis and statistical diagram showing the proportion of CD4^+^ and CD8^+^ T cells in tumors.

In vivo experiments showed no significant change in the tumorigenic capacity of PLA2G7 knockdown cells in immunodeficient mice (Fig. 4A, B). However, the tumorigenic capacity of PLA2G7 knockdown cells was markedly impaired in immunocompetent mice (Fig. 4C, D). FACS detection of transplanted tumors indicated a significant increase in the proportion of CD8+ T cells in PLA2G7 knockdown tumors (Fig. 4E). Detection of T cell activity factors also revealed significantly increased T cell activity in PLA2G7 knockdown tumors (Supplementary Fig. S2). Based on these results, we suggest that PLA2G7 can promote PD-L1 expression and immune evasion in bladder cancer.

### PLA2G7 regulates the JAK–STAT pathway through the phosphorylations of STAT1 and STAT3

**Fig. 5:**
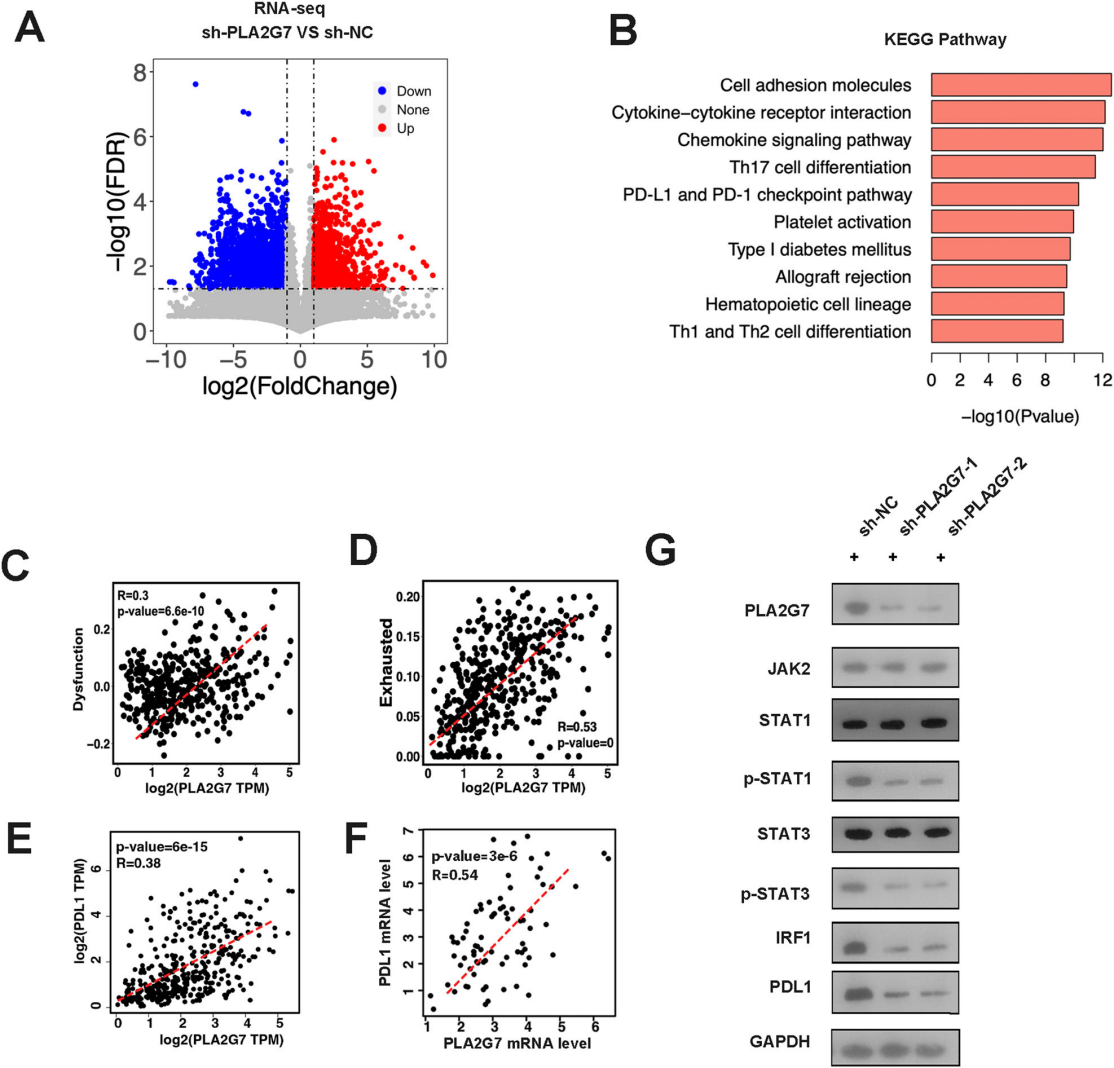
PLA2G7 regulates the JAK–STAT pathway through the phosphorylations of STAT1 and STAT3. **A** Volcano plot of genes differentially expressed in sh-NC and sh-PLA2G7 cells following stimulation with IFN-γ. **B** KEGG enrichment analysis for significant down-regulated genes. **C** The correlation of PLA2G7 with dysfunction score in TCGA bladder cancer cohort. **D** The correlation of PLA2G7 with exhausted score in TCGA bladder cancer cohort. **E** The correlation of PLA2G7 with PD-L1 mRNA level in TCGA bladder cancer cohort. **F** The correlation of PLA2G7 with PD-L1 expression in bladder cancer tissues analyzed by RT-qPCR. **G** Western blot analysis showing PLA2G7, JAK2, STAT1, p-STAT1, STAT3, p-STAT3, IRF1 and PD-L1 levels in sh-NC and sh-PLA2G7 cells.

We next examined the phenotypic changes in PLA2G7 knockdown cells at the global transcriptome level. Using DEseq2, we identified 3343 differentially expressed genes, including 1297 upregulated and 2046 downregulated genes (Fig. 5A). KEGG pathway analysis showed that these genes were mainly associated with cell adhesion, cytokine receptor interaction, chemokine signaling, and several immune-related pathways (Fig. 5B). Further analysis revealed that PLA2G7 expression was positively associated with immune dysfunction scores calculated by Tumor Immune Dysfunction and Exclusion (TIDE) and exhausted scores calculated by ImmunecellAI (Fig. 5C, D). Consistently, PLA2G7 was positively correlated with PD-L1 expression in the TCGA cohort and RT-qPCR results (Fig. 5E, F). The JAK-STAT-IRF-PD-L1 pathway is one of the most important pathways regulating PD-L1 expression in tumors. We then examined the effect of PLA2G7 knockdown on this pathway and found that the protein levels of p-STAT1, p-STAT3, and IRF1 were significantly decreased in PLA2G7 knockdown cells compared to control cells with IFN-γ stimulation (Fig. 5G).

### PLA2G7 inhibitor darapladib suppresses bladder cancer immune evasion

**Fig. 6:**
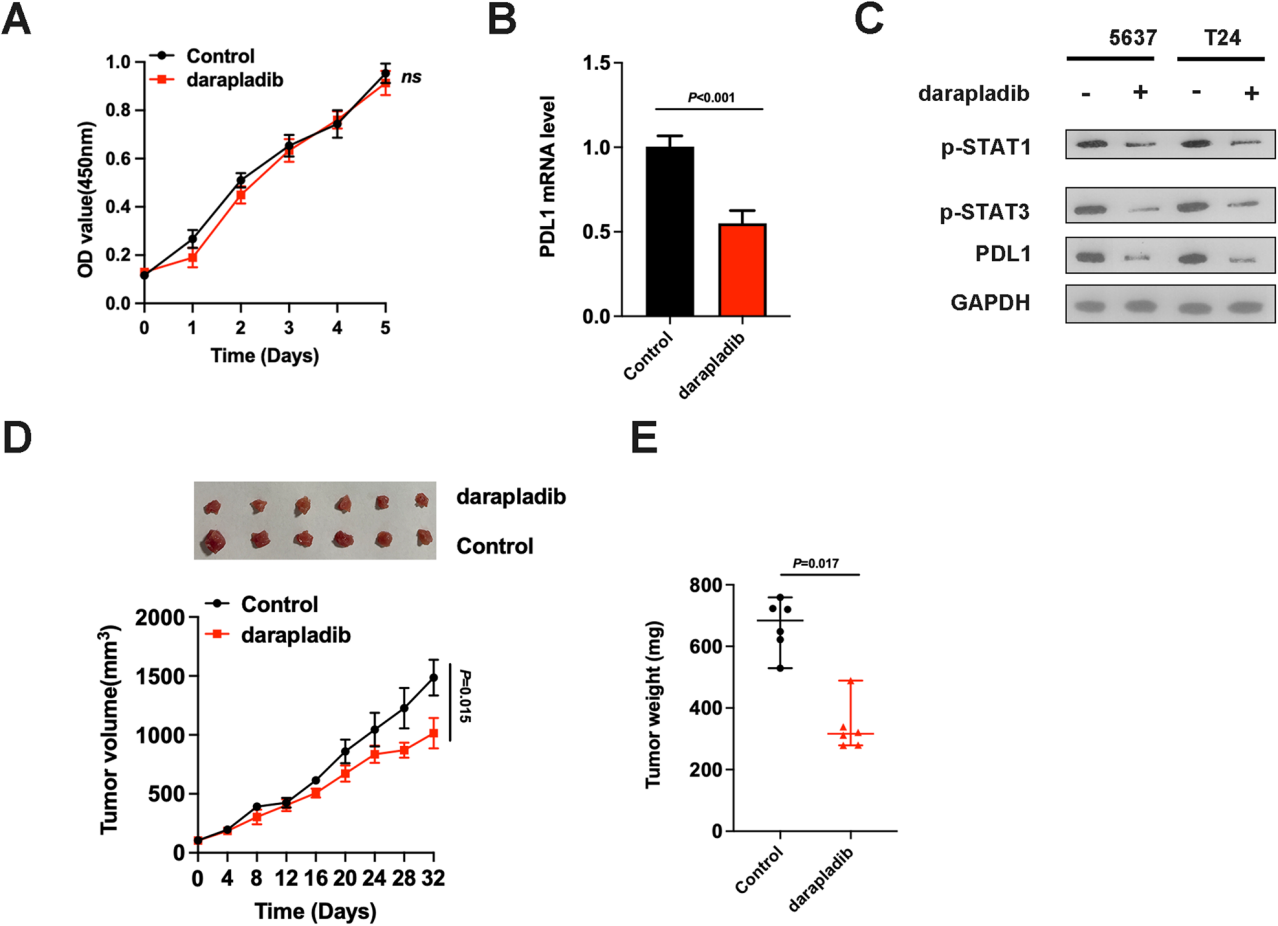
PLA2G7 inhibitor darapladib suppresses bladder cancer immune evasion. **A** MTS assays of 5637 cell viability treated with placebo and darapladib. **B** RT-qPCR analysis of the mRNA levels of PD-L1 in bladder cancer cells treated with placebo and darapladib. **C** Western blot analysis showing p-STAT1, p-STAT3 and PD-L1 levels in bladder cancer cells treated with placebo and darapladib. **D** Tumor growth curves for immunocompetent BALB/C mice injected with bladder cancer cells treated with placebo and darapladib. **E** Tumor weights for immunocompetent BALB/C mice injected with bladder cancer cells treated with placebo and darapladib.

We further investigated the effect of the PLA2G7 inhibitor darapladib on bladder cancer cells. MTS assays showed that darapladib had no significant effect on the proliferation of bladder cancer cells (Fig. 6A). However, PD-L1 mRNA levels significantly decreased after treatment with darapladib (Fig. 6B). The protein levels of p-STAT1, p-STAT3, and PD-L1 also significantly decreased after treatment with darapladib (Fig. 6C). Treatment with the PLA2G7 inhibitor darapladib significantly impaired the tumorigenic capacity of bladder cancer cells in immunocompetent mice (Fig. 6D, E).

### PLA2G7 overexpression promotes immune evasion of bladder cancer cells

**Fig. 7:**
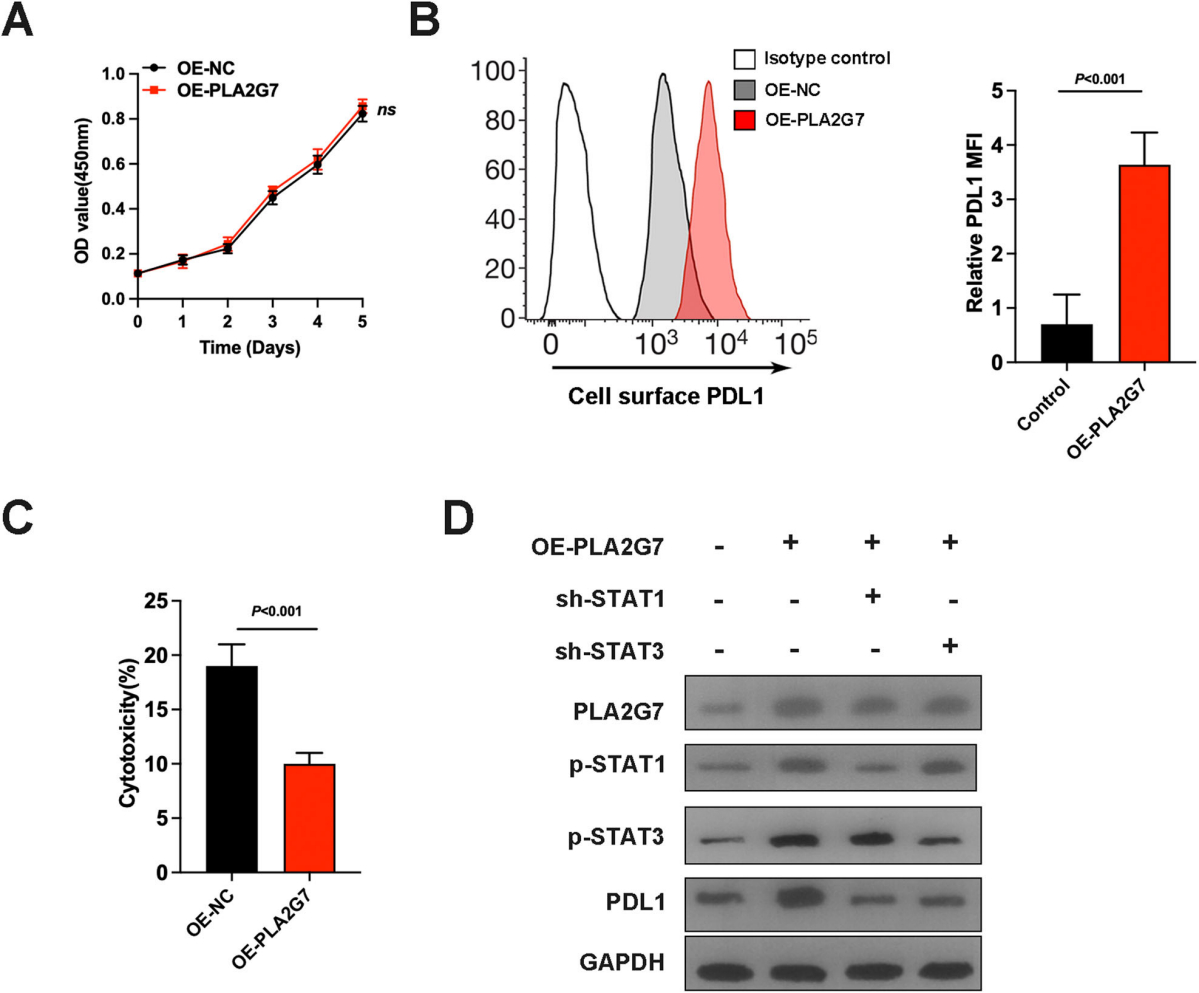
PLA2G7 overexpression promotes PD-L1 expression and tumor immune evasion. **A** MTS cell viability assay of OE-NC and OE-PLA2G7 cells. **B** Surface PD-L1 protein levels analyzed by FACS in OE-NC and OE-PLA2G7 cells. **C** Results from the T cell killing assays of OE-NC and OE-PLA2G7 cells. **D** Western blot analysis of PLA2G7, p-STAT1, p-STAT3 and PD-L1 levels in OE-NC and OE-PLA2G7 cells with or without STAT1 and STAT3 knockdown.

We then constructed bladder cancer cells with stable overexpression of PLA2G7. MTS assays revealed no significant difference in cell proliferation capacity (Fig. 7A). Using FACS assays, we found that overexpression of PLA2G7 significantly promoted PD-L1 expression (Fig. 7B). T cell co-culture experiments demonstrated that overexpression of PLA2G7 significantly reduced T cell cytotoxicity (Fig. 7C). Meanwhile, overexpression of PLA2G7 increased the expression levels of p-STAT1, p-STAT3, and PD-L1. Knockdown of STAT1 and STAT3 significantly inhibited the promoting effect of PLA2G7 overexpression on PD-L1 expression (Fig. 7D).

### PLA2G7 depletion in combination with CTLA4 blockade effectively suppresses tumor growth

**Fig. 8:**
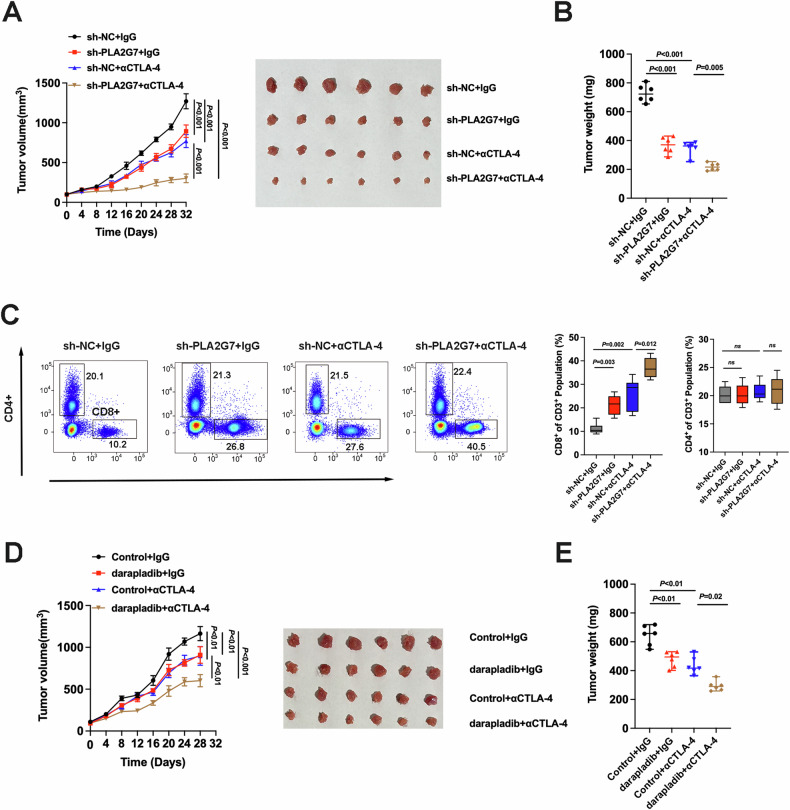
PLA2G7 inhibition sensitizes the efficacy of CTLA4 blockade. **A** Tumor growth of MBT-2 cells with stable PLA2G7 depletion in BALB/C mice with or without anti-CTLA4 antibody treatment. **B** Comparative analysis of tumor weight in Fig. 8A. **C** Flow cytometry analysis and statistical plot showing the proportion of CD4^+^ and CD8^+^ T-cells in the tumors. **D** Tumor growth of MBT-2 cells in BALB/C mice treated with darapladib and anti-CTLA4 antibody. **E** Comparative analysis of tumor weight in Fig. 8D.

Since anti-PD-L1 and anti-CTLA4 immunotherapies utilize different mechanisms, their combined application has a synergistic effect to enhance the effectiveness of immunotherapy. Our results suggested that PLA2G7 depletion inhibits PD-L1 expression in bladder cancer cells. We further explored the synergistic effect between PLA2G7 depletion and CTLA4 blockade in mouse model experiments. BALB/C mice were inoculated with PLA2G7-depleted or control MBT-2 cells and then treated with either control or anti-CTLA4 antibody when tumors were palpable. Results indicated that PLA2G7 depletion reduced MBT-2 tumor growth, and the combination of PLA2G7 depletion with CTLA4 blockade significantly improved the inhibitory effect on tumor burden (Fig. 8A, B). The combination also improved mouse survival (Supplementary Fig. S3A).

Combination treatment of PLA2G7 depletion and anti-CTLA4 antibody significantly promoted CD8+ T cell infiltration compared to CTLA4 blockade alone (Fig. 8C). Flow cytometry analysis of mouse tumors revealed that PLA2G7 depletion elevated T cell activation, and the combination of PLA2G7 depletion with CTLA4 blockade significantly improved T cell activation, as assessed by the levels of TNF-α, IFN-γ, and GZMB (Supplementary Fig. S3B). Treatment with the PLA2G7 inhibitor darapladib combined with CTLA4 blockade also significantly inhibited the growth of transplanted tumors in mice (Fig. 8D, E). Collectively, these data suggest that PLA2G7 depletion has the potential to enhance the efficacy of anti-CTLA4 antibody.

### ETS1 promotes the expression of PLA2G7 and immune evasion of bladder cancer

**Fig. 9:**
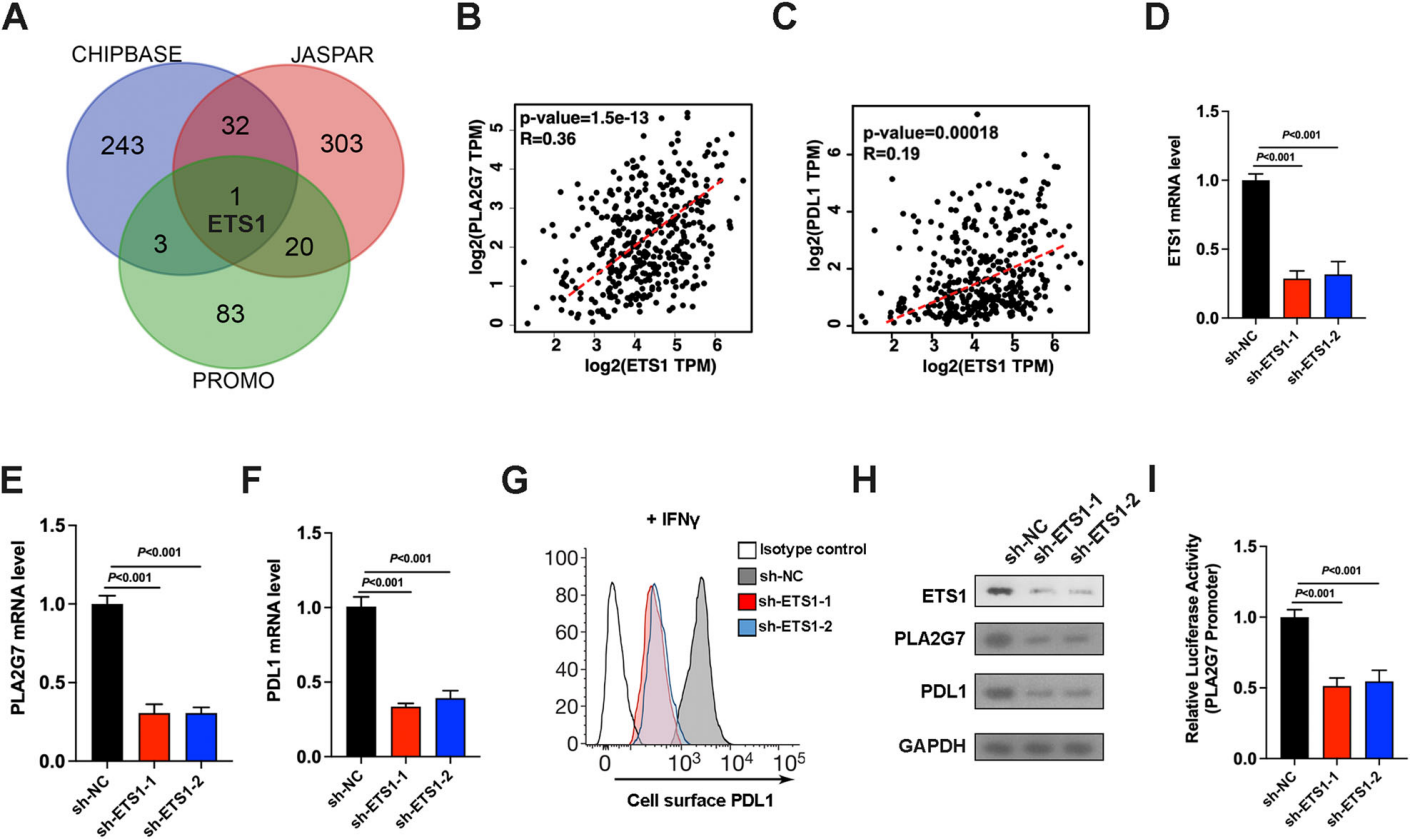
Transcription factor ETS1 regulates the expression of PLA2G7. **A** In silico analysis of potential transcription factors in PLA2G7 promoter region. **B** The correlation of PLA2G7 with ETS1 expression in bladder cancer tissues from the TCGA database. **C** The correlation of PD-L1 with ETS1 expression in bladder cancer tissues from the TCGA database. **D** RT-qPCR analysis of ETS1 mRNA levels in sh-NC and sh-ETS1 cells. **E** RT-qPCR analysis of PLA2G7 mRNA levels in sh-NC and sh-ETS1 cells. **F** RT-qPCR analysis of PD-L1 mRNA levels in sh-NC and sh-ETS1 cells. **G** Surface PD-L1 levels were analyzed by FACS in sh-NC cells and sh-ETS1 cells. **H** Western blot analysis showing ETS1, PLA2G7, and PD-L1 levels in sh-NC and sh-ETS1 cells. **I** Luciferase reporter assays of PLA2G7 promoter in sh-NC and sh-ETS1 cells.

To elucidate the mechanisms underlying PLA2G7 overexpression in bladder cancer, we initially examined genomic alterations, including copy number variations (CNVs) and methylation status of PLA2G7 within TCGA bladder cancer data. However, these analyses yielded negative results. Subsequently, we conducted in silico analyses using publicly available databases (ChIPBase, JASPAR, and PROMO) to identify potential cis-regulatory elements within the PLA2G7 promoter region (spanning from −2000 bp to the transcription start site). By integrating the findings from these approaches, we identified ETS1 as a potential transcription factor for PLA2G7 (Fig. 9A). Further analysis of TCGA expression data revealed a significant positive correlation between ETS1 and PLA2G7 as well as PD-L1 expression in bladder cancer tissues (Fig. 9B, C).

To investigate the functional role of ETS1 in regulating PLA2G7 expression, we established bladder cancer cell lines with stable ETS1 knockdown (Fig. 9D). Quantitative RT-PCR revealed a significant decrease in PLA2G7 mRNA levels in ETS1 knockdown cells compared to control cells (Fig. 9E). Similarly, PD-L1 mRNA levels were significantly reduced in PLA2G7 knockdown cells (Fig. 9F). Flow cytometry analysis demonstrated that ETS1 knockdown significantly reduced PD-L1 expression (Fig. 9G). Western blot analysis confirmed that the expression of ETS1, PLA2G7, and PD-L1 was significantly downregulated in ETS1 knockdown cells (Fig. 9H). Additionally, luciferase reporter assays showed that ETS1 shRNAs significantly downregulated PLA2G7 promoter activity (Fig. 9I). Collectively, these findings indicate that ETS1 positively regulates PLA2G7 expression in bladder cancer, thereby contributing to immune evasion.

## Discussion

The latest data show that bladder cancer is the seventh most common cancer globally and the fourth most common cancer in males [1]. Despite advances in surgical techniques and chemotherapy, the prognosis for patients with bladder cancer remains poor, especially for those with advanced and metastatic disease [4]. In recent years, cancer immunotherapy has made significant strides in treating bladder cancer due to ongoing research into tumor immune evasion mechanisms. One of the major mechanisms of tumor immune evasion is the activation of the PD-1/PD-L1 pathway, which inhibits T-cell proliferation and activation, ultimately leading to immune evasion by tumor cells and promoting tumor development and metastasis. Therefore, the PD-1/PD-L1 pathway is considered a highly promising target for cancer immunotherapy. Several anti-PD-1/PD-L1 drugs have been approved for the treatment of advanced bladder cancer and have demonstrated acceptable efficacy [25]. However, due to tumor heterogeneity, PD-L1 expression regulation, and the tumor microenvironment, only a subset of bladder cancer patients benefits from PD-1/PD-L1 immunotherapy. PD-L1 expression is regulated by various transcription factors, including STAT1, HIF1/2, STAT3, IRFs, and NFκB [26, 27]. The regulatory mechanisms of PD-L1 expression in bladder cancer have not yet been fully elucidated, and exploring these mechanisms is crucial for developing potential therapeutic strategies and improving immunotherapy efficacy. At the same time, recent studies have also continuously discovered new regulatory mechanisms that play important roles in the immune evasion and progression, as well as metastasis of bladder cancer [28–31].

Phospholipase A2 group VII (PLA2G7) is a phospholipase involved in the hydrolysis of platelet-activating factor and truncated phospholipids synthesized through oxidation [32]. In recent years, PLA2G7 has been extensively studied in the context of cardiovascular diseases, with the PLA2G7 inhibitor darapladib being investigated for the prevention and treatment of coronary heart disease [33–35]. Meanwhile, recent studies have shown that PLA2G7 is highly expressed in various tumors and can serve as a prognostic factor and therapeutic target. In colon tumorigenesis, high PLA2G7 expression has been proposed to play a causal role, and PLA2G7 deletion has been shown to decrease intestinal polyposis and colon tumor formation in Apc^Min/+^ mice [15, 36]. In breast cancer, PLA2G7 has been identified as a regulator of the Wnt signaling pathway and hormone receptor negativity, as well as a factor involved in epithelial-mesenchymal transition [19, 20]. PLA2G7 has also been reported as a novel prognostic factor in prostate cancer and a potential therapeutic target through apoptosis regulation [17, 18, 37, 38]. Silencing PLA2G7 expression in large B cell lymphoma cell lines has been shown to impede proliferation and migration while inducing apoptotic death, with comparable results observed using the PLA2G7 inhibitor darapladib [16]. Recent investigations have highlighted the potential of PLA2G7 as a promising biomarker with diagnostic and prognostic implications across diverse malignancies [14, 16, 39]. In hepatocellular carcinoma, PLA2G7-high macrophages represent a highly immunosuppressive subset that hinders CD8+ T-cell activation, suggesting PLA2G7 as a key factor in tumor immune regulation [23].

In this study, we found that PLA2G7 is overexpressed in bladder cancer and significantly associated with worse prognosis. In vitro experiments suggested that PLA2G7 knockdown does not significantly affect bladder cancer cell proliferation, migration, or invasion. Further analysis revealed that PLA2G7 depletion significantly inhibits PD-L1 expression in bladder cancer cells and suppresses tumor growth in vivo. We found that the IFN-γ pathway is significantly affected by PLA2G7 knockdown, with STAT1 and STAT3 identified as downstream genes. Knocking down PLA2G7 significantly inhibited the phosphorylation of STAT1 and STAT3. Notably, combining PLA2G7 depletion with CTLA-4 blockade significantly improved the suppression of tumor burden and mouse survival rates. We also demonstrated that the transcription factor ETS1 promotes PLA2G7 expression in bladder cancer.

In summary, our study suggests that ETS1-mediated overexpression of PLA2G7 can regulate the phosphorylation of STAT1 and STAT3, leading to PD-L1 overexpression and promoting immune evasion in bladder cancer.

## Supplementary information

Supplemental figures

Western Blot

Supplementary Tables

## Data Availability

The datasets used and/or analyzed during the current study are available from the corresponding author upon reasonable request. TCGA bladder cancer expression data and clinical data were downloaded from UCSC Xena (https://xenabrowser.net/heatmap/).
