# Supplementary material for: PLA2G7 promotes immune evasion of bladder cancer through the JAK-STAT-PDL1 axis
Source: Cell Death Dis. 2025 Apr 1;16(1):234. doi: 10.1038/s41419-025-07593-1 (PMC11962123; doi:10.1038/s41419-025-07593-1)

**Figure S1:**

Representative images illustrating the effects of PLA2G7 knockdown on cell migration (A) and invasion(B).


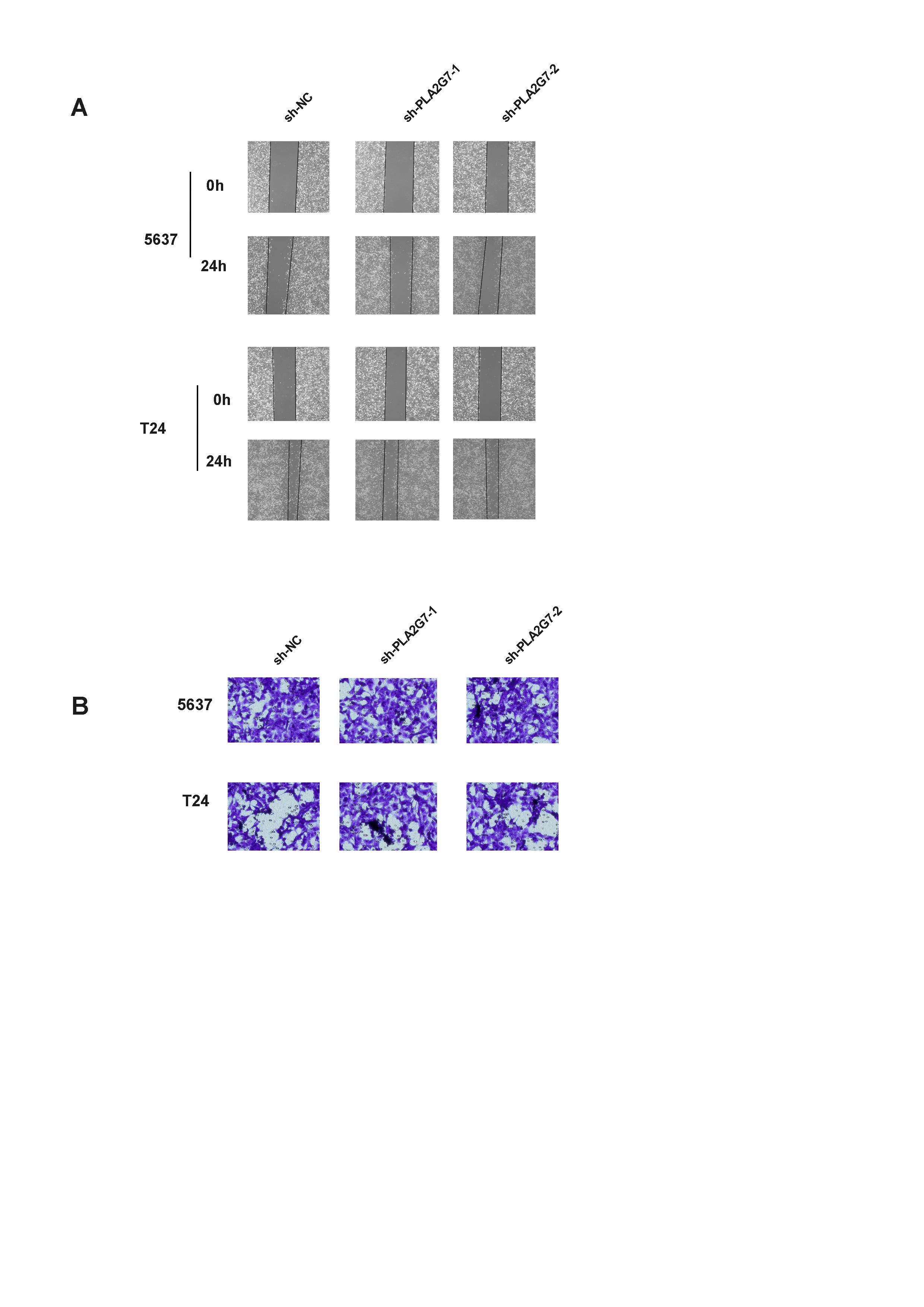


**Figure S2:**

T cell activity factor TNFα, IFN-γ, and GZMB in sh-NC and sh-PLA2G7 tumors analyzed by Flow cytometry.


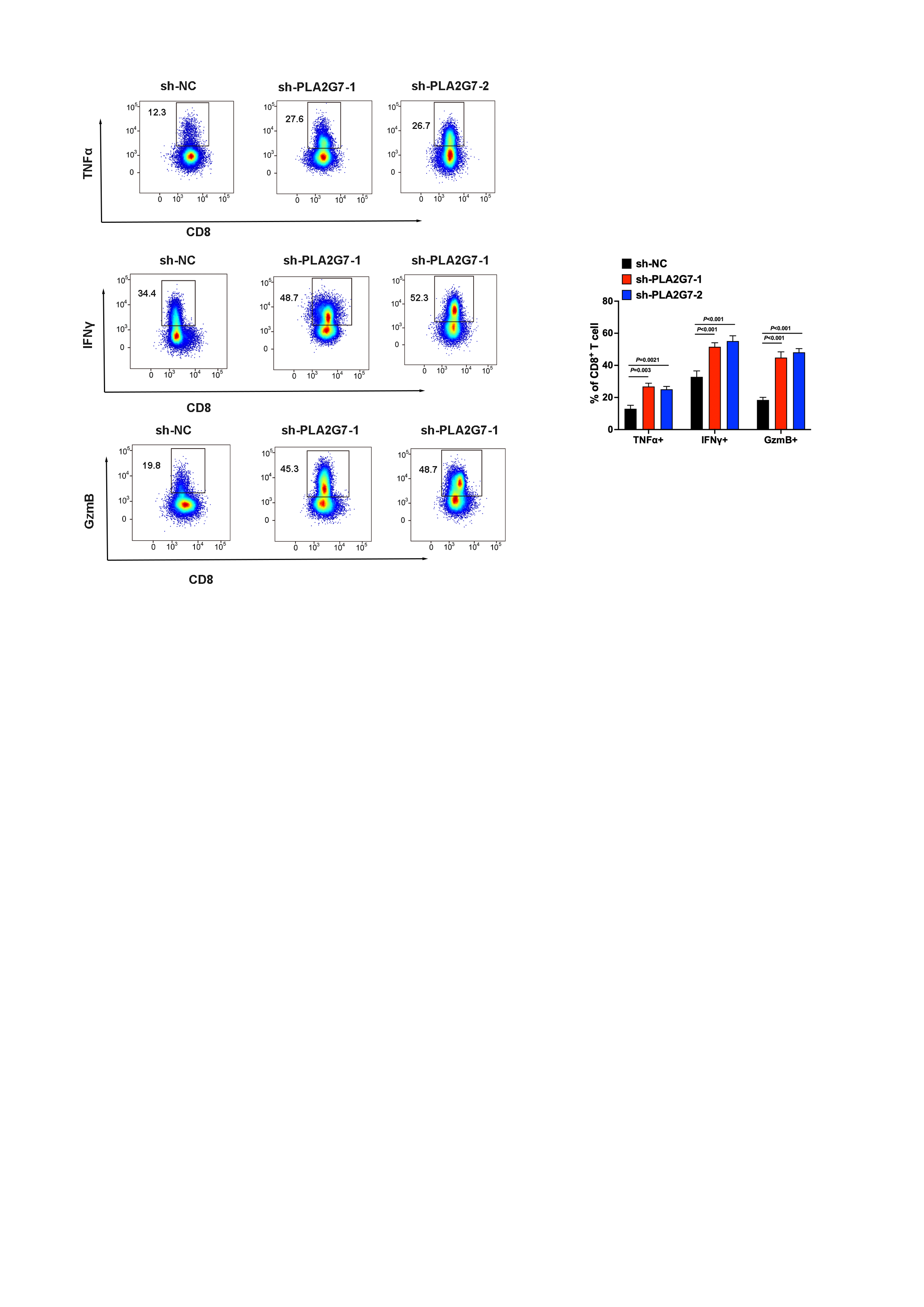


**Figure S3:**

(A) The survival curve of BALB/C mice with sh-NC and sh-PLA2G7 cells treated with or without anti-CTLA-4 antibody.

(B) T cell activity factor TNFα, IFN-γ, and GZMB analyzed by Flow cytometry in MBT-2 tumors with stable PLA2G7 depletion in BALB/C mice after treatment with or without anti-CTLA-4 antibody.


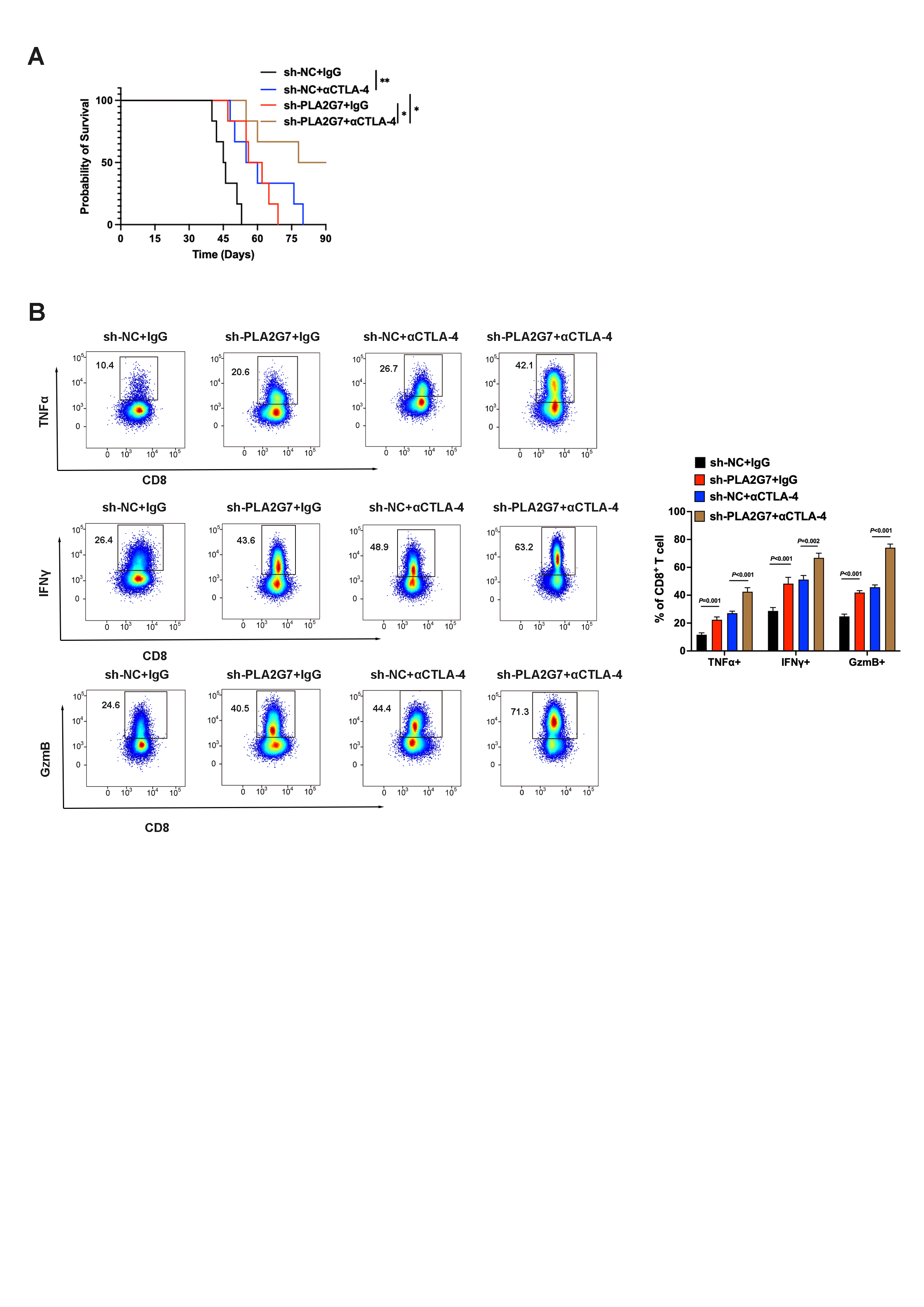

Supplement: Supplementary file 1 — Supplemental figures [file 41419_2025_7593_MOESM1_ESM.docx]
