# Supplementary material for: PLA2G7 promotes immune evasion of bladder cancer through the JAK-STAT-PDL1 axis
Source: Cell Death Dis. 2025 Apr 1;16(1):234. doi: 10.1038/s41419-025-07593-1 (PMC11962123; doi:10.1038/s41419-025-07593-1)

Figure 1 C PLA2G7 tissues


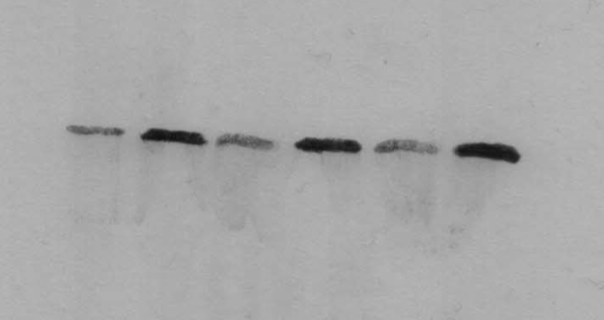


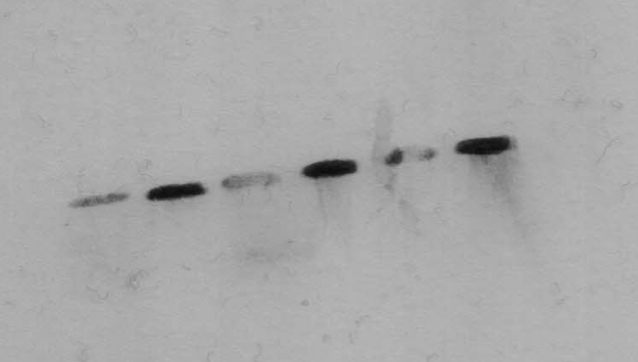


Figure 1 C GAPDH tissues


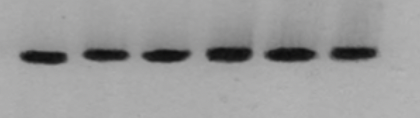


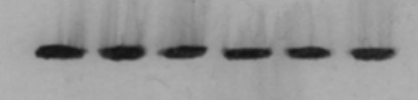


Figure 1 C PLA2G7 cell lines


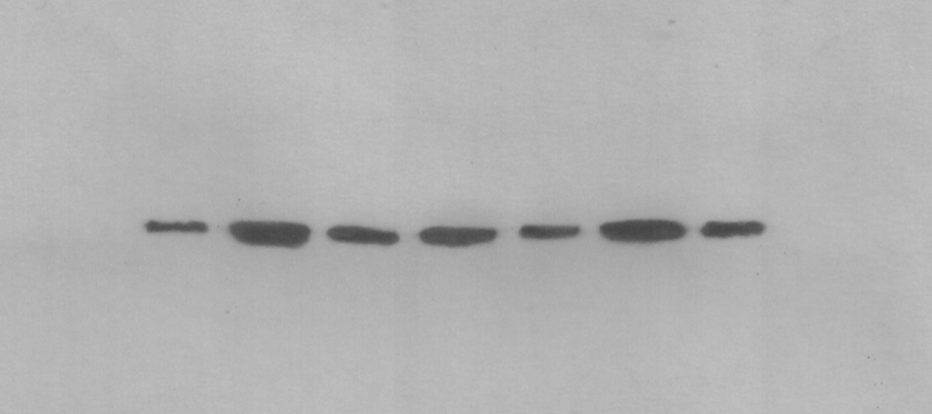


Figure 1 C GAPDH cell lines


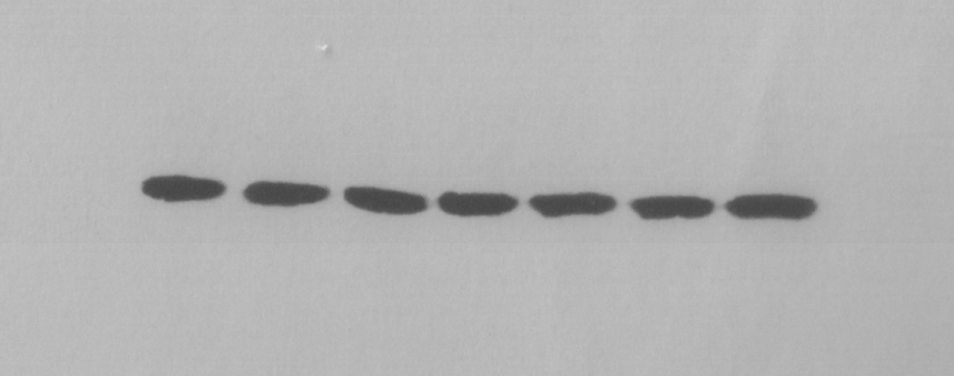


Figure 2A PLA2G7-5637


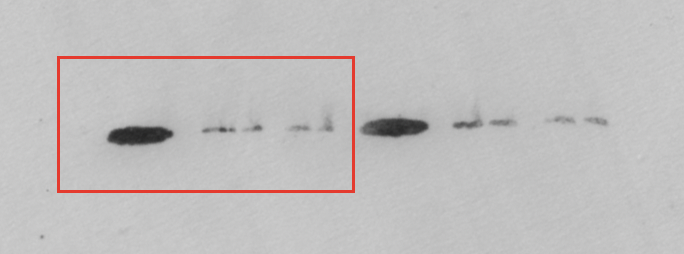


Figure 2A GAPDH-5637


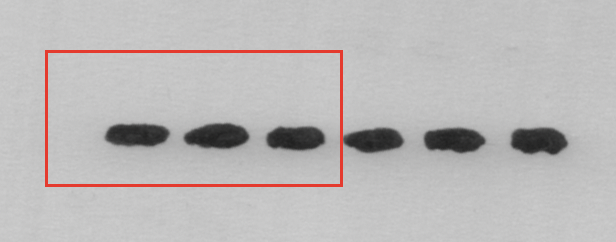


Figure 2A PLA2G7-T24


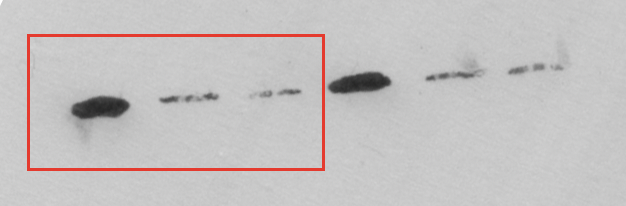


Figure 2A GAPDH-T24


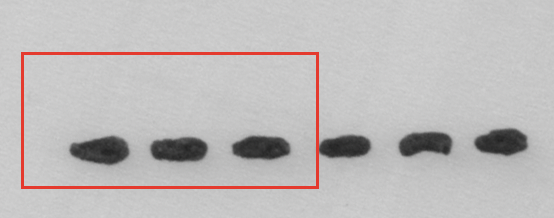


Figure 3B PLA2G7


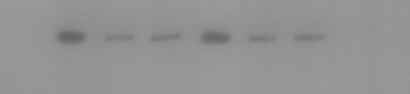


Figure 3B PDL1


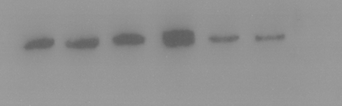


Figure 3B GAPDH


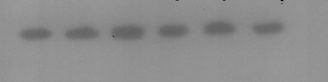


Figure 5G PLA2G7


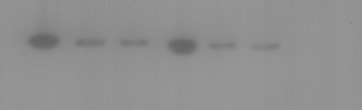


Figure 5G JAK2


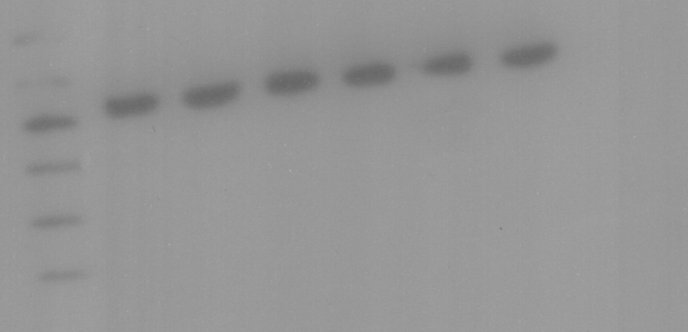


Figure 5G STAT1


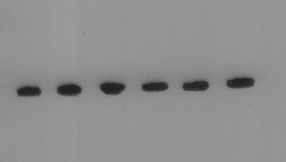


Figure 5G P-STAT1


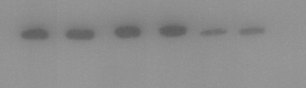


Figure 5G STAT3


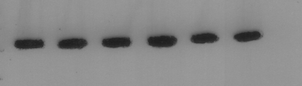


Figure 5G P-STAT3


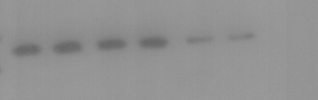


Figure 5G IRF1


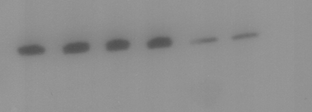


Figure 5G PDL1


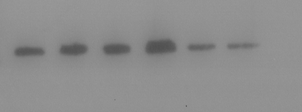


Figure 5G GAPDH


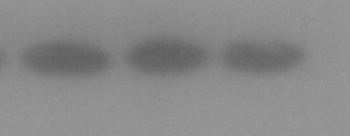


Figure6 C p-STAT1


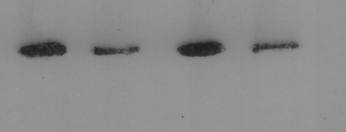


Figure6 C P-STAT3


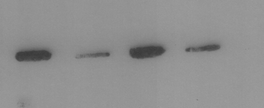


Figure6 C PDL1


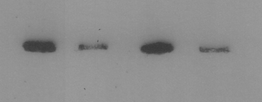


Figure6 C GAPDH


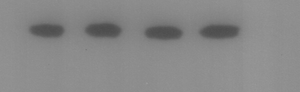


Figure 7D PLA2G7


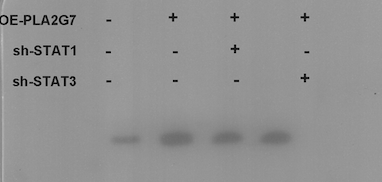


Figure 7D P-STAT1


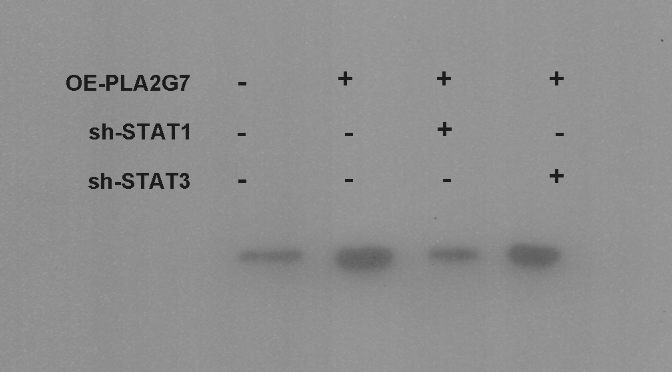


Figure 7D P-STAT3


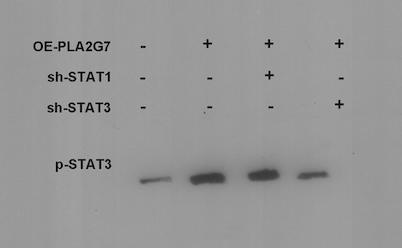


Figure 7D PDL1


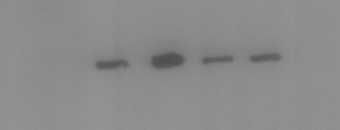


Figure 7D GAPDH


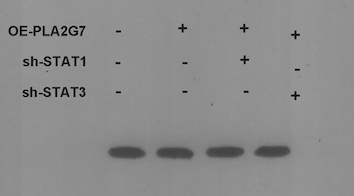


Figure 9F ETS1


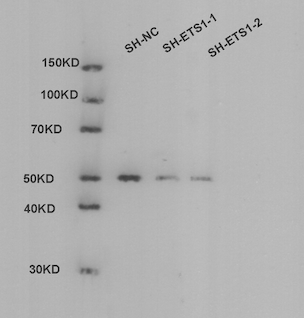


Figure 9F PLA2G7


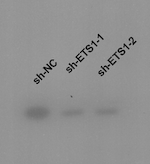


Figure 9F PDL1


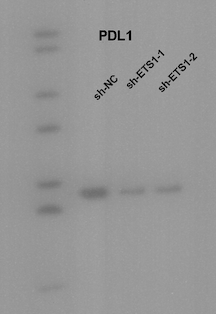


Figure 9F GAPDH


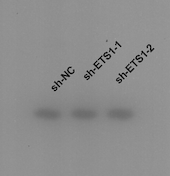

Supplement: Supplementary file 2 — Western Blot [file 41419_2025_7593_MOESM2_ESM.docx]
