# Supplementary material for: PLA2G7 promotes immune evasion of bladder cancer through the JAK-STAT-PDL1 axis
Source: Cell Death Dis. 2025 Apr 1;16(1):234. doi: 10.1038/s41419-025-07593-1 (PMC11962123; doi:10.1038/s41419-025-07593-1)
Supplement: Supplementary file 3 — Supplementary Tables [file 41419_2025_7593_MOESM3_ESM.docx]

Supplementary Table 1. Characteristics of the corresponding shRNA sequences included in the context.

| shRNA | Sequence (5' to 3") |
| --- | --- |
| shPLA2G7-#1 | GCTCAAATTAAAGGGAGACAT |
| shPLA2G7-#2 | CGTTGGTTGTACAGACTTAAT |
| shETS1-#1 | CTGGAATTACTCACTGATAAA |
| shETS1-#2 | GCCCTGGGTAAAGACTGCTTT |
| shSTAT1 | CCCTGAAGTATCTGTATCCAA |
| shSTAT3 | GCTGACCAACAATCCCAAGAA |
| mouse-shPLA2G7-#1 | CGTTTGTACTACCCAGCTCAA |
| mouse-shPLA2G7-#2 | CGGAAAGAACAGGTTCAGCAA |

Supplementary Table 2. Characteristics of the corresponding qPCR primers included in the context.

| Genes | Forward primer | Reverse primer |
| --- | --- | --- |
| PLA2G7 | TAAGTTAACCGCGGGTCCAG | AGCACATGCAATTTGGGTGG |
| PDL1 | GCTGCACTAATTGTCTATTGGG | CACAGTAATTCGCTTGTAGTCG |
| GAPDH | CAAATTCCATGGCACCGTCA | GACTCCACGACGTACTCAGC |
| mouse-PLA2G7 | TCACAAGCTCCAATCGGTGAT | CGACGGGGTACGATCCATTTC |
| mouse-PDL1 | TGCGGACTACAAGCGAATCACG | CTCAGCTTCTGGATAACCCTCG |
| mouse-GAPDH | AGGTCGGTGTGAACGGATTTG | TGTAGACCATGTAGTTGAGGTCA |
